# From perceived risk to action: an individual-level study of how self-determination and new media shape public emergency rescue participation in China

**DOI:** 10.3389/fpsyg.2025.1645770

**Published:** 2025-12-04

**Authors:** Runhan Zhang

**Affiliations:** Department of Justice, Shaanxi Police College, Xi’an, Shaanxi, China

**Keywords:** Self-Determination Theory, personal emergency risk perception, collective protective consciousness, social belongingness, new media engagement effect

## Abstract

**Introduction:**

Grounded in Self-Determination Theory, this study examines the mechanisms shaping individuals’ willingness to join grassroots emergency rescue teams. The research focuses on how psychological motivation and risk cognition jointly influence participation in emergency response behaviors.

**Methods:**

A total of 428 valid survey responses were collected, and Structural Equation Modeling (SEM) was used to test an integrative framework consisting of psychological factors, emergency risk perception, and behavioral intention. Moderation and mediation effects were analyzed to clarify the pathways of influence.

**Results:**

Collective protective consciousness, social belongingness, and emergency response self-efficacy significantly reduced individuals’ emergency risk perception. Elevated risk perception, in turn, decreased willingness to participate in emergency rescue. Emergency risk perception partially mediated the effects of the three psychological factors on behavioral intention. Additionally, new media engagement moderated the relationship between risk perception and willingness to participate—higher engagement weakened the inhibitory effect of risk on intention.

**Discussion:**

The findings expand the application of Self-Determination Theory to the context of disaster volunteerism and demonstrate how risk perception functions as both a psychological barrier and a motivational converter. The results highlight the importance of fostering psychological readiness and leveraging new media platforms to enhance volunteer mobilization and emergency preparedness.

## 1 Introduction

Against the backdrop of escalating global climate change, the frequent occurrence of extreme natural disasters has become a major threat to the security of human societies (Islam and Khan, 2020). According to the United Nations report The Human Cost of Disasters: 2000–2019, China is among the countries most severely affected by natural disasters, with increasing pressure on disaster prevention and mitigation in recent years. During the 2021 Henan torrential rains, grassroots emergency rescue teams demonstrated a rapid response capacity, accounting for 37% of total rescue operations (Lu et al., 2024), signaling a shift in the role of civil society forces from “supplementary” to “collaborative” in disaster response. However, there remain bottlenecks in coordination between the official rescue system and social forces. On one hand, public participation channels are limited, and social resource integration is insufficient (Booher and Innes, 2005); on the other hand, information asymmetry leads to cognitive biases in public risk perception, affecting participation decisions (Pilemalm, 2018). The traditional “government-led–public-passive” emergency response model (Loukis and Charalabidis, 2015) is increasingly inadequate for complex disaster scenarios, calling for theoretical innovation to identify effective pathways for enhancing public participation.

Against this backdrop, new media has emerged as a crucial platform for risk communication and public mobilization. Its diverse interactive mechanisms—including information acquisition, emotional resonance, and social connection—have been shown to significantly influence public risk perception in disaster contexts. Studies indicate that during disasters, new media functions not only as a channel for information dissemination but also as a means of emotional relief, trust building, and fostering collective identity. Especially in the aftermath of major events, its role tends to shift toward bidirectional interaction and relationship construction (Lambert, 2020). For instance, during the COVID-19 pandemic, public risk perception and emotional expression exhibited a dynamic coupling through platforms such as Weibo and search engines, with new media amplifying both the speed of information dissemination and the intensity of emotional responses (Hou et al., 2020). However, a clear theoretical framework is still lacking on how the effects of new media moderate the pathway between risk perception and willingness to engage in emergency response.

This study centers on the core path of “psychological motivation–risk perception–behavioral intention” to construct a research model and fill the theoretical and empirical gaps in pre-disaster mobilization research. First, guided by Self-Determination Theory (SDT), it integrates the complete pathway among intrinsic motivation, risk perception, and participation intention, offering a new theoretical perspective for understanding the psychological mechanisms behind public emergency behavior. Second, the study introduces Collective Protective Consciousness as a key group-level variable to examine how individuals’ strong identification with group responsibility, public safety, and social goals facilitates the internalization of motivation and enhances their willingness to participate in emergency rescue. Lastly, it systematically analyzes the moderating role of Emergency Response Self-Efficacy—including information acquisition, emotional resonance, and social interaction—on the relationship between Personal Emergency Risk Perception and Intention to Join Emergency Response Teams, thereby providing empirical support for media intervention strategies and public mobilization pathways.

To clearly define the study purpose and ensure consistency with the proposed conceptual framework, this research establishes one overarching objective and four sub-objectives. The primary objective of this study is to examine how self-determination mechanisms and new media engagement jointly influence the public’s intention to participate in emergency rescue activities in China.

To achieve this goal, the study is guided by the following sub-objectives:

1. To analyze how Collective Protective Consciousness, Social Belongingness, and Emergency Response Self-Efficacy affect individuals’ Personal Emergency Risk Perception.
2. To verify the mediating role of Personal Emergency Risk Perception between psychological motivation variables and the Intention to Join Emergency Response Teams.
3. To explore how the New Media Engagement Effect moderates the relationship between Personal Emergency Risk Perception and Intention to Join Emergency Response Teams.
4. To propose theoretical and managerial implications for improving public engagement in grassroots emergency rescue through the lens of Self-Determination Theory (SDT) and digital media mobilization.

Through these objectives, the study aligns its analytical framework with five independent variables—Collective Protective Consciousness, Social Belongingness, Emergency Response Self-Efficacy, Personal Emergency Risk Perception, and New Media Engagement Effect—and one outcome variable, Intention to Join Emergency Response Team. This alignment strengthens the coherence between the research objectives, hypotheses, and analytical model.

## 2 Theoretical framework and hypotheses

### 2.1 Self-Determination Theory

Self-Determination Theory (SDT), proposed by Deci and Ryan in the 1980s, serves as a macro-theoretical framework for studying human motivation and has become one of the most influential motivation theories across disciplines (Deci and Ryan, 2013). Rooted in organismic dialectical philosophy, the theory identifies three basic psychological needs—competence, autonomy, and relatedness—and posits that the degree to which these needs are satisfied directly influences individuals’ motivation, psychological wellbeing, and social adaptability (Ryan and Deci, 2000). Based on this foundation, Cognitive Evaluation Theory systematically explains how external events influence intrinsic motivation by affecting perceptions of autonomy and competence (Deci and Ryan, 2000). In addition, Organismic Integration Theory transcends the dichotomy between intrinsic and extrinsic motivation by proposing a continuum of internalization of extrinsic motivation, incorporating external regulation, introjected regulation, identified regulation, and integrated regulation within the SDT framework (Deci et al., 2017).

Applications of Self-Determination Theory (SDT) across various disciplines—including psychology, education, organizational behavior, and public health—have demonstrated its interdisciplinary integration potential and universal applicability. In the field of disaster volunteer research, numerous scholars have explored the topic from different perspectives. For example, Prytz et al. (2023) and Gonzalez-Mendez conducted studies focusing on volunteers. The former, based on a survey of 5,347 volunteers, found that community connectedness, self-image, and perceived competence were positive predictors of continued volunteer service, whereas alert fatigue and negative experiences served as negative predictors. The latter, through a study on volunteers from the Spanish Red Cross, highlighted that self-compassion and self-determination contribute to distinguishing different psychological states among volunteers, playing a significant role in preventing adverse psychological outcomes. Other scholars have focused on theoretical development. For instance, Wu and Li (2019), based on SDT, found that a supportive work environment influences volunteer motivation, which in turn affects emotional exhaustion and life satisfaction. Fernandes and de Matos (2023) confirmed the critical roles of basic psychological needs and value congruence in promoting volunteer participation and value co-creation behaviors. These studies provide multidimensional theoretical support and practical guidance for understanding volunteer behavior and improving volunteer service management.

This study is grounded in Self-Determination Theory (SDT) and explores the psychological mechanisms underlying the willingness of grassroots emergency rescue team members to participate. Social belongingness reflects individuals’ sense of affiliation through identification with group relationships and shared goals, which helps to foster prosocial behavior and the intention to engage in volunteer activities (Pavey et al., 2011). Personal emergency risk perception corresponds to the dimension of competence; when individuals believe they are capable of handling disaster-related tasks, their motivation to act is enhanced (Edmunds et al., 2006). Collective protective consciousness represents an internalized sense of social responsibility, aligning with the “integrated regulation” level of SDT, in which individuals transform external social norms into internalized values and proactively act upon them (Haivas et al., 2013).

Although SDT has been widely applied in studies of volunteer behavior, existing literature primarily focuses on the direct pathways between basic psychological needs and intrinsic motivation (Messmann et al., 2022), with limited exploration of how external situational factors influence behavioral intentions through psychological mechanisms. In high-pressure social contexts such as disaster response, individuals are not solely driven by need satisfaction but are also influenced by their subjective assessment of external risks. Therefore, this study introduces personal emergency risk perception as a mediating variable to address the theoretical gap concerning the role of external context in shaping motivation and intention within the SDT framework.

Emergency risk perception refers to individuals’ subjective psychological state in response to external disaster scenarios. It influences how individuals interpret the fulfillment of psychological needs and determines whether such interpretations translate into a willingness to participate. Studies have shown that when personal emergency risk perception is excessively high and individuals perceive themselves as lacking the necessary capabilities, they may withdraw from action despite having altruistic motivation, due to anxiety and a heightened sense of threat (Najafi et al., 2022). Conversely, when emergency risk perception is at a moderate level and individuals have sufficient self-confidence in their ability to respond, it can enhance their sense of responsibility and self-efficacy, thereby positively affecting their willingness to engage in disaster response (Cai et al., 2023). This effect is particularly evident among emergency responders, whose willingness to act significantly increases when high risk awareness is accompanied by strong self-efficacy (Kang et al., 2023).

Moreover, emergency risk perception functions not only as a warning mechanism but also as a form of psychological arousal that influences how individuals interpret experiences related to social belonging and competence satisfaction. As such, it constitutes a dynamic psychological chain from psychological needs → risk perception → intrinsic motivation → behavioral intention. This perspective not only addresses the limitations of previous SDT applications that emphasized internal mechanisms while overlooking the role of external perceptions, but also better reflects the actual dynamics of volunteer behavior in high-risk social contexts.

In summary, this study incorporates personal emergency risk perception into the SDT framework as a mediating variable between psychological needs, intrinsic motivation, and participation intention. It seeks to explore the mechanism emphasized by SDT regarding how external environments influence internal motivation.

### 2.2 Collective protective consciousness

Collective protective consciousness reflects an individual’s proactive willingness to safeguard common interests and public safety. Its connotation is deeply embedded in the synergistic mechanisms of social capital, risk perception, and group interaction. Studies have shown that the core components of social capital—trust, norms, and networks—can enhance group coordination by reinforcing these elements (Su et al., 2021), encouraging individuals to regard personal safety and collective wellbeing as inseparable (Lalot et al., 2021). This consciousness is not only manifested in shared alertness to potential risks, but also in a sense of proactive responsibility rooted in systems thinking and critical awareness (Maker Castro et al., 2022), whereby individuals transcend personal interests in crisis situations to form a psychological contract to protect collective safety (Orianne and Eustache, 2023).

This psychological contract is expressed through the promotion of collective action in emergency response via shared cognitive frameworks (Su et al., 2021), and through emotional bonds that strengthen individual identification with the collective (Cheng et al., 2021). The aforementioned studies confirm that collective protective consciousness is essentially the product of the dynamic interaction between individual agency and the broader social system (Sakdiyakorn et al., 2021).

### 2.3 Personal emergency risk perception

Personal emergency risk perception refers to an individual’s cognitive judgment regarding potential threats and their impact on personal safety in the face of sudden incidents. It encompasses a multidimensional evaluation of event severity, controllability, and uncertainty (Shen et al., 2021). Research indicates that such perceptions are influenced not only by personal experiences and emotional responses (Wachinger et al., 2013), but also by levels of social trust and the channels through which information is accessed (Chan et al., 2020). Within a collective context, information sharing and resource integration among members foster the perception that “the group possesses sufficient information and resources to cope with potential risks” (Orianne and Eustache, 2023), which strengthens individuals’ sense of risk controllability and thereby reduces personal emergency risk perception.

Collective protective consciousness can also give rise to a diffusion of responsibility effect, in which individuals perceive that they share risk responsibilities with other group members, thus alleviating the psychological burden of perceived risk (Su et al., 2021). Moreover, collective protective consciousness encourages individuals to participate in coordinated collective action, fostering a belief that “risks can be effectively managed.” This belief further reduces individual personal emergency risk perception, as individuals internalize collective interests as personal interests (Cheng et al., 2021), shifting their attention toward the overall risk status of the group and away from excessive focus on personal risk.

Based on the above analysis, this study proposes that stronger collective protective consciousness corresponds to weaker perceived individual risk in the face of emergencies, and a lower degree of concern about potential threats.

*H1:* Collective protective consciousness is negatively associated with personal emergency risk perception.

### 2.4 Social belongingness

Social belongingness, as a key expression of emotional connection and identification between individuals and groups, describes a psychological state in which individuals feel accepted, valued, and recognized within social relationships (Pardede and Kovač, 2023). It serves not only as a protective factor for psychological adjustment, but also as a major motivator driving individuals to engage in collective action and respond to crises. Studies indicate that social belongingness encompasses not only the cognitive recognition of group membership (Lee and Robbins, 1995), but also the process through which individuals establish self-identity in social environments via emotional support or value contribution. For instance, social-value representation has been found to significantly enhance the fulfillment of individuals’ need for belonging (Pardede and Kovač, 2023).

Further research reveals that the absence of belongingness may intensify psychological risks. A lack of belongingness can amplify the impact of peer victimization on depressive symptoms (Roberts et al., 2021), while a strong sense of family belongingness can effectively mitigate suicidal ideation (Parra et al., 2021). In the context of collective action, such as disaster recovery, a sense of belonging functions as a core element in strengthening individuals’ social bonds, facilitating resilience and sustainable recovery (Tennakoon and Serrao-Neumann, 2021), thereby reducing individual anxiety when facing risks.

Based on this analysis, the study proposes that a higher level of social belongingness can help alleviate individuals’ anxiety and concern when confronted with potential threats.

*H2:* Social belongingness is negatively associated with personal emergency risk perception.

### 2.5 Emergency response self-efficacy

Emergency response self-efficacy, as a core concept of social cognitive theory, refers to an individual’s multidimensional cognitive assessment of their own ability to perform emergency tasks in the context of a sudden incident. It reflects a dynamic cognitive mechanism formed through past experience, vicarious learning, and emotional feedback (Bandura, 1997). In the field of emergency management, this construct manifests as confidence in risk response skills, such as performing rescue operations (Labrague et al., 2021). Its significance lies in its direct influence on individuals’ decision-making, level of effort, and persistence in emergency participation.

According to existing research, individuals with high self-efficacy are more likely to take proactive disaster preparedness measures (Gammoh et al., 2022), and by enhancing the accuracy of risk perception, they promote household emergency readiness (Wang et al., 2021). This efficacy belief not only strengthens decision-making capabilities in complex disaster environments but also buffers the negative effects of stress on emergency actions. Its formation involves multiple pathways, including knowledge acquisition, skill proficiency, and positive feedback loops (Labrague et al., 2021; Wang et al., 2021).

Based on the above analysis, the need for autonomy drives individuals to actively acquire emergency knowledge. This self-determined behavior enhances their perceived competence and forms a positive cycle for risk management. Therefore, this study argues that the higher an individual’s emergency response self-efficacy, the lower their subjective perception of emergency risk.

*H3:* Emergency response self-efficacy is negatively associated with personal emergency risk perception.

### 2.6 Intention to join emergency response team

Intention to join an emergency response team reflects the public’s motivational tendency to proactively assume social responsibility during unexpected events. Its core connotation involves the combined effects of individuals’ subjective assessment of emergency risks, self-efficacy beliefs, and emotional-social connectedness (Chen et al., 2022). Research has shown that personal emergency risk perception is a key antecedent driving participation in emergency rescue. When individuals recognize the potential threat posed by a risk to themselves or society, they are more likely to develop the intention to intervene (Agha, 2003).

In emergency decision-making, personal emergency risk perception directly influences behavioral tendencies. The importance of intention to join an emergency response team lies in its role as a psychological intermediary between objective risk and subjective behavior (Wahlberg and Sjoberg, 2000). Especially in crisis contexts, high levels of risk perception may enhance individuals’ reliance on collective action, yet may also trigger a “risk perception paradox,” whereby individuals choose to act despite awareness of the danger (Wachinger et al., 2013).

Existing research indicates that risk perception comprises three dimensions: concern about immediate harm, judgment of consequence severity, and anxiety regarding uncertainty (Shen et al., 2021), all of which influence the intention to join an emergency response team. Therefore, this study proposes the following hypothesis:

*H4:* Personal emergency risk perception is negatively associated with intention to join emergency response team.

### 2.7 Mediating effect

Collective protective consciousness reflects individuals’ sense of responsibility toward the collective and their psychological readiness for coordinated action. In essence, it constitutes an inward transformation of “social mobilization capacity.” This consciousness not only reduces individuals’ subjective fear of disaster threats (Su et al., 2021), but also facilitates the formation of a controllable risk judgment through pathways such as information sharing, responsibility distribution, and cognitive integration (Orianne and Eustache, 2023).

Such positive risk assessments further enhance individuals’ sense of security in their environment and stimulate their motivation to act, making them more willing to undertake coordinated response tasks. As a result, personal emergency risk perception decreases. In this mechanism, personal emergency risk perception plays a transitional role: on one hand, it originates from collective protective consciousness, which strengthens perceptions of risk controllability; on the other hand, it releases the psychological energy needed to convert protective awareness into actual willingness to participate. Therefore, the following hypothesis is proposed:

*H5:* Personal emergency risk perception mediates the relationship between collective protective consciousness and intention to join emergency response team.

Social belongingness reflects individuals’ emotional connection and value identification with the group, and serves as a critical form of social capital that drives behavioral mobilization under crisis conditions (Pardede and Kovač, 2023). When individuals receive acceptance, trust, and emotional support within a group, their sense of social value is enhanced, and their anxiety about sudden events is alleviated (Tennakoon and Serrao-Neumann, 2021).

In this context, social belongingness strengthens perceived social support and trust in group resources, fostering the belief that one is “not facing risks alone” when confronted with potential threats. This shift in risk assessment effectively reduces individuals’ personal emergency risk perception. The resulting change in perception serves as a psychological trigger for greater willingness to engage in emergency response actions. Therefore, the following hypothesis is proposed:

*H6:* Personal emergency risk perception mediates the relationship between social belongingness and intention to join emergency response team.

Emergency response self-efficacy determines whether individuals perceive themselves as “capable” of handling disaster situations (Bandura, 1997), and this belief profoundly influences their subjective risk assessment. Individuals with high emergency response self-efficacy tend to believe they can manage complex situations, resulting in lower levels of risk perception (Labrague et al., 2021; Wang et al., 2021). This reduced perception of risk helps individuals avoid excessive anxiety, thereby enhancing their psychological preparedness and motivation to act.

In other words, self-efficacy operates through a mediating pathway involving risk perception, enabling the stable activation of motivation and its translation into behavioral intention. Therefore, the following hypothesis is proposed:

*H7:* Personal emergency risk perception mediates the relationship between emergency response self-efficacy and intention to join emergency response team.

### 2.8 New media engagement effect

The new media engagement effect refers to the systematic influence of individuals’ interactive behaviors—such as information acquisition, emotional resonance, and mobilization for action—through social media and online platforms on their cognition, emotions, and behavioral intentions. During emergency events, new media serves as a primary channel for disseminating risk-related information. It not only shapes individuals’ perceptions of the severity of the event but also guides public risk interpretation and behavioral judgment through the sharing of peer experiences and the spread of both positive and negative emotions (Chan et al., 2020; Wachinger et al., 2013).

Related studies suggest that new media affects individual behavioral intention on two levels: first, through frequent exposure to disaster scenarios and volunteer action content, it raises public awareness of social responsibility and stimulates the desire to participate in rescue efforts (Kim and Hastak, 2018); second, due to the emotional amplification effect inherent in the information ecology of new media, individuals may experience heightened fear or anxiety, resulting in excessive risk perception that ultimately suppresses actual participation (Wang et al., 2021).

Based on the above logic, the new media engagement effect may serve as a moderator in the relationship between personal emergency risk perception and intention to join an emergency response team. Specifically, if the information disseminated through new media emphasizes narratives of risk controllability and effective coping, it may weaken the negative impact of high risk perception on participation intention. Conversely, if the content highlights disaster threats, lacks actionable guidance, or creates a panic-inducing atmosphere, it may amplify individuals’ negative emotional responses to risk, causing them to withdraw or hesitate despite having initial motivation.

As a situational variable, the new media engagement effect may alter the psychological pathway through which subjective risk perception is translated into behavioral intention, thus acting as a “moderator of the risk perception–action conversion mechanism.” Based on this reasoning, the following hypothesis is proposed:

*H8:* The new media engagement effect moderates the relationship between personal emergency risk perception and intention to join emergency response team.

**FIGURE 1:**
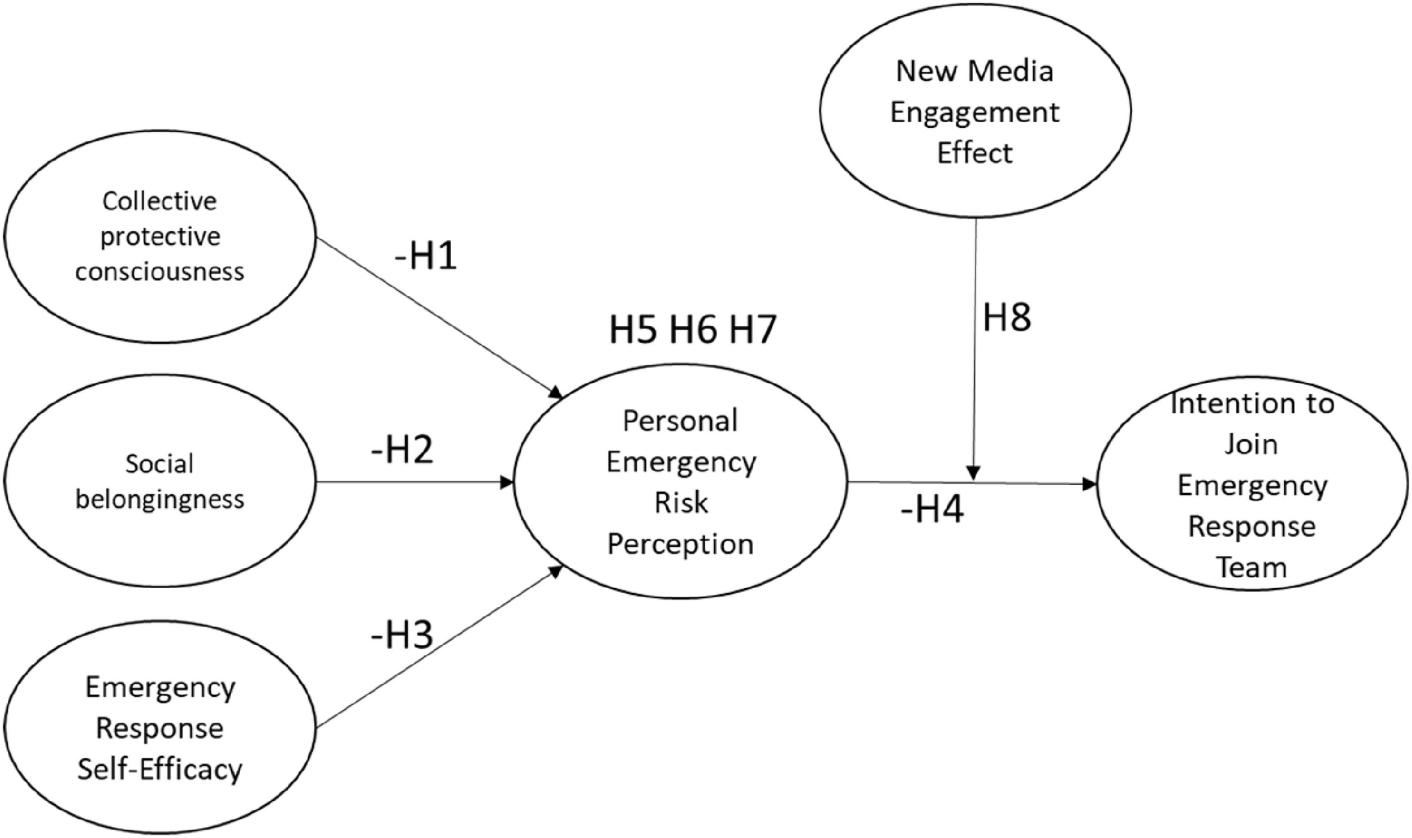
Research framework.

This study aims to reveal the mechanisms through which collective protective consciousness, social belongingness, and emergency response self-efficacy influence individuals’ intention to join an emergency response team. It further explores the mediating role of personal emergency risk perception and the moderating role of the new media engagement effect. The proposed research framework is illustrated in Figure 1.

## 3 Research design

### 3.1 Research participants and data collection

This study adopts a quantitative cross-sectional research design. This study targeted members of the general public in China to examine the factors influencing individuals’ willingness to participate in emergency rescue activities. The target population of this study consisted of adult residents aged 18–65 from several major provinces in mainland China, including Beijing, Shanghai, Guangdong, and Hubei. These areas were selected because they represent diverse levels of urbanization and socioeconomic development. A stratified convenience sampling approach was employed to ensure representation across gender, age, and occupational groups. To ensure the representativeness of the sample, the minimum required size was determined using the Survey System sample size calculator, based on a 95% confidence level and a 5% margin of error, resulting in a minimum of 384 respondents. The formal data collection was conducted between June and December 2024. A total of 442 questionnaires were distributed through both online (QR code scanning) and offline (paper-based) channels, and 428 valid responses were obtained after excluding incomplete or invalid questionnaires, yielding an effective response rate of 97% (428/442 × 100). All participants provided informed consent prior to completing the survey. The data collection process adhered to ethical research standards, ensuring participant anonymity and voluntary participation. The collected data were subsequently analyzed using Structural Equation Modeling (SEM) through SmartPLS 4.0.

### 3.2 Description of research variables

The proposed model includes one outcome variable—Intention to Join Emergency Response Team; three independent variables—Collective Protective Consciousness, Social Belongingness, and Emergency Response Self-Efficacy; one mediating variable—Personal Emergency Risk Perception; and one moderating variable—New Media Engagement Effect. To clarify the constructs used in this study, the operational definitions of all variables are provided in detail below.

In addition, individual demographic characteristics, including gender, age, and occupation, were included as control variables. All measurement items used in this study were adapted from well-established scales published in reputable SSCI and SCI journals. A rigorous translation–back translation procedure was adopted to ensure the accuracy and cultural equivalence of the Chinese versions of the scales.

All variables were measured using a seven-point Likert scale, ranging from 1 (strongly disagree) to 7 (strongly agree). A higher score indicates a greater degree of agreement with the corresponding construct. The specific measurement design of each variable is detailed as follows:

#### 3.2.1 Collective protective consciousness

Collective protective consciousness emphasizes individuals’ sense of identification with the collective, responsibility, and proactive protective behaviors. Li (2021), in the study “A New Look at the Racial Differences in Environmental Attitudes: The Roles of Risk Perception and Group Consciousness,” examined the impact of group consciousness on environmental attitudes and participatory behaviors. The concept of group consciousness is highly relevant to collective protective consciousness, as the latter essentially represents group members’ sense of responsibility and willingness to safeguard shared interests. In Li’s study, the Cronbach’s alpha for the four items used to measure group consciousness was 0.84, indicating a high level of internal consistency. Based on the operationalization in this study and aligned with the concept of collective protective consciousness, the present research developed seven items for measurement.

#### 3.2.2 Social belongingness

Social belongingness emphasizes acceptance, inclusion, and connectedness. This study draws on the General Belongingness Scale (GBS) developed by Malone et al. (2012), published in Personality and Individual Differences. The scale was designed to measure achieved belongingness and was validated through exploratory and confirmatory factor analyses (EFA and CFA), identifying two core dimensions: acceptance/inclusion and rejection/exclusion, which align with the conceptual foundation of social belongingness. The overall Cronbach’s alpha for the GBS was 0.94, indicating excellent internal consistency. The acceptance/inclusion factor specifically yielded a Cronbach’s alpha of 0.92, further confirming its measurement reliability. Based on the structure of the GBS and consistent with the conceptualization of social belongingness, this study developed the six items.

#### 3.2.3 Emergency response self-efficacy

Emergency response self-efficacy emphasizes confidence in rescue actions, the application of emergency knowledge, collaboration with others, and decision-making during emergency situations. This study draws on the work of Harp et al. (2017), titled “Volunteer Engagement and Retention: Their Relationship to Community Service Self-Efficacy,” which examined the influence of community service self-efficacy on volunteer engagement and retention. The study provided relevant measurement items, and the overall Cronbach’s alpha for the Community Service Self-Efficacy Scale was reported at 0.94, indicating excellent internal consistency. The Volunteer Engagement Scale showed a Cronbach’s alpha of 0.91, further supporting the reliability of the instrument. Following the measurement approach of the Community Service Self-Efficacy Scale and adapting it to the concept of emergency response self-efficacy, this study developed the six items.

#### 3.2.4 Personal emergency risk perception

Personal emergency risk perception refers to individuals’ multidimensional subjective judgment of risk during emergency situations, formed through the evaluation of exposure susceptibility, consequence severity, information uncertainty, perceived effectiveness of protective measures, and social impact. This study developed a measurement scale for personal emergency risk perception based on the work of Shen et al. (2021), titled “Development and Psychometric Assessment of the Public Health Emergency Risk Perception Scale: Under the Outbreak of COVID-19.” The original scale measured risk perception across three dimensions—fear, severity, and unknown—which provide a contextualized and multidimensional framework suitable for this study’s emergency context. The original scale demonstrated acceptable reliability, with a Cronbach’s alpha of 0.793. In this study, the items were adapted through scenario-specific revisions while retaining the original scale’s validity. Five items were ultimately developed.

#### 3.2.5 New media engagement effect

New media engagement effect refers to individuals’ multidimensional subjective judgment formed during emergency situations through information acquisition, interactive participation, perceived trust, perceived efficacy, and social connectedness via new media platforms. This study developed a measurement scale for new media engagement effect based on the study by Tang et al. (2022), titled “The Bright Side of Digitization: Assessing the Impact of Mobile Phone Domestication on Left-Behind Children in China’s Rural Migrant Families.” In that study, the Cronbach’s alpha for the scale measuring new media use among left-behind children was 0.82, indicating good reliability. The measurement incorporated three dimensions: Information connectivity, emotional support, and behavioral participation. The framework of “technologically mediated social connection” is applicable to emergency situations. Based on the conceptual framework and item structure from Tang et al., this study developed five adapted items.

#### 3.2.6 Intention to join emergency response team

Intention to join an emergency response team refers to an individual’s subjective tendency and plan to participate in volunteer emergency rescue services within a future time frame. It reflects the individual’s intentional judgment regarding the behavior and focuses specifically on the strength of the decision to engage in such actions. This study adapted its measurement items from Warburton and Terry (2018), whose work “Volunteer Decision Making by Older People: A Test of a Revised Theory of Planned Behavior” applied the Theory of Planned Behavior to examine the intention dimension of volunteer participation. The Cronbach’s alpha for the intention construct in their study was 0.84, indicating high internal consistency and strong reliability and validity. Based on this framework and in alignment with the construct of intention to join an emergency response team, the following four items were developed.

### 3.3 Method of analysis

This study employed Structural Equation Modeling (SEM) for data analysis. All questionnaire items were measured using a seven-point Likert scale, which meets the statistical assumption of normal distribution. The data analysis was conducted in three stages: descriptive statistical analysis, measurement model validation, and structural model evaluation. This study adopts a quantitative cross-sectional design. A total of 442 questionnaires were distributed, and 428 valid responses were collected, yielding an effective response rate of 97% (428/442 × 100). All analyses were conducted using SmartPLS 4.0. All eight hypotheses (H1–H8) proposed in this study were tested simultaneously within a single Structural Equation Modeling (SEM) framework using SmartPLS 4.0. Both the measurement model and the structural model were estimated based on the same dataset. This unified analytical approach ensures methodological consistency and theoretical coherence across all hypotheses, confirming that no separate analytical tools were employed for hypothesis testing. In the first stage, SPSS was used to perform descriptive statistical analysis of the objective variables in the study, such as gender, age, and occupation. This stage consisted of two steps: first, calculating the frequency distribution of demographic data; second, computing the mean and standard deviation for each variable. In the second stage, confirmatory analyses were conducted through SEM to evaluate the measurement and structural models. Reliability analysis, convergent validity, and discriminant validity were used to validate the measurement model. Finally, based on the proposed research model, SmartPLS 4.0 was used to test the research hypotheses.

The structural equation model used in this study can be expressed as follows:


Y=β+0βCPC1+βSB2+βSE3+βPE4+βNM5+ε

where Y represents the Intention to Join Emergency Response Team, CPC denotes Collective Protective Consciousness, SB is Social Belongingness, SE is Emergency Response Self-Efficacy, PE is Personal Emergency Risk Perception, and NM is New Media Engagement Effect. All estimated coefficients (β), standard deviations, *t*-values, and *p*-values are summarized in Table 1. In the narrative discussion, only the significance levels (*p* < 0.05) are emphasized together with the interpretation of each variable’s directional influence.

**TABLE 1:** Results of path analysis.

Hypothesized path	Path coefficient (β)	SD	*T*-value	*P*-value
Collective protective consciousness→personal emergency risk perception	–0.287	0.048	5.990	0.000
Emergency response self-efficacy→personal emergency risk perception	–0.310	0.047	6.653	0.000
Social belongingness→personal emergency risk perception	–0.259	0.050	5.202	0.000
Personal emergency risk perception→intention to join emergency response team, intention to JERT	–0.368	0.051	7.164	0.000

## 4 Research results

### 4.1 Demographic profile and descriptive statistical analysis

#### 4.1.1 Demographic profile of respondents

A total of 428 valid responses were collected. The gender distribution was nearly balanced, with 213 male respondents (49.8%) and 215 female respondents (50.2%). In terms of age, 186 respondents (43.5%) were under the age of 35, 161 (37.6%) were between 36 and 55 years old, and 81 (18.9%) were over 56. Regarding occupation, the majority of respondents were employed in the private sector or self-employed, accounting for 195 individuals (45.6%). This was followed by those working in state-owned or centrally administered enterprises (76 individuals, 17.8%), freelancers (51 individuals, 11.9%), public institution employees (49 individuals, 11.4%), civil servants (39 individuals, 9.1%), and active-duty or retired military personnel (18 individuals, 4.2%). These results indicate that the sample covers a wide range of demographic backgrounds, ensuring the diversity and representativeness necessary for subsequent analysis, as shown in Table 2.

**TABLE 2:** Demographic profile of respondents.

Category	Group	Frequency	Percentage (%)
Gender	Male	213	49.8
	Female	215	50.2
Age	35 and below	186	43.5
	36–55	161	37.6
	Above 56	81	18.9
Occupation	Freelancer	51	11.9
	Public institution employee	49	11.4
	Civil servant	39	9.1
	State-owned/central enterprise	76	17.8
	Private sector/self-employed	195	45.6
	Active-duty/retired military	18	4.2

#### 4.1.2 Descriptive statistical analysis

The data in this study meet the basic assumptions for multivariate statistical analysis. Descriptive statistics show that the mean values for the Personal Emergency Risk Perception (PE) group ranged between 2.710 and 2.740, while the means for the other groups ranged from 5.150 to 5.330, indicating notable intergroup differences. Skewness test results revealed that most variables exhibited mild to moderate negative skewness (approximately –0.904 to –0.611), whereas the PE group demonstrated a positive skewness pattern (approximately 0.769–0.880). Kurtosis values overall fell within the range of –0.411 to 0.204. According to Kline (2023), when the absolute value of skewness is < 2 and the absolute value of kurtosis is < 7, the data can be considered to meet the assumptions of conventional statistical methods. In addition, the standard deviations for all variables ranged from 1.441 to 1.571, indicating an acceptable level of dispersion. Taken together—based on skewness, kurtosis, and variability—this dataset satisfies the requirements for model suitability in statistical analysis, as shown in Table 3.

**TABLE 3:** Descriptive statistics of study variables.

Item	Mean	SD	Skewness	Kurtosis
CPC1	5.250	1.488	–0.833	0.051
CPC2	5.250	1.441	–0.666	–0.257
CPC3	5.330	1.460	–0.694	–0.176
CPC4	5.240	1.491	–0.831	0.153
CPC5	5.260	1.469	–0.771	0.086
CPC6	5.290	1.466	–0.824	0.021
CPC7	5.220	1.463	–0.801	0.141
SB1	5.200	1.491	–0.710	–0.170
SB2	5.220	1.494	–0.702	–0.043
SB3	5.160	1.457	–0.647	–0.102
SB4	5.230	1.488	–0.620	–0.370
SB5	5.180	1.490	–0.622	–0.232
SB6	5.180	1.449	–0.651	–0.083
SE1	5.280	1.549	–0.863	–0.099
SE2	5.270	1.518	–0.849	0.204
SE3	5.250	1.507	–0.844	0.147
SE4	5.250	1.556	–0.819	–0.037
SE5	5.310	1.496	–0.904	0.187
SE6	5.250	1.485	–0.772	–0.002
PE1	2.710	1.571	0.866	–0.135
PE2	2.720	1.535	0.822	–0.081
PE3	2.710	1.539	0.769	–0.242
PE4	2.720	1.509	0.870	0.151
PE5	2.740	1.560	0.880	0.134
NM1	5.200	1.499	–0.690	–0.247
NM2	5.200	1.498	–0.691	–0.153
NM3	5.180	1.486	–0.738	–0.052
NM4	5.150	1.480	–0.620	–0.359
NM5	5.160	1.532	–0.611	–0.411
JE1	5.320	1.537	–0.808	–0.103
JE2	5.270	1.553	–0.824	0.106
JE3	5.250	1.485	–0.754	–0.020
JE4	5.300	1.538	–0.814	0.068

CPC, collective protective consciousness; SB, social belongingness; SE, emergency response self-efficacy; PE, personal emergency risk perception; NM, new media engagement effect; JE, intention to join emergency response team.

### 4.2 Convergent validity

The convergent validity of the measurement model was assessed based on the framework proposed by Fornell and Larcker (1981) and the psychometric standards of Nunnally (1994). Four core indicators were used for the validation: (1) standardized factor loadings of latent variables ( ≥ 0.70), (2) composite reliability (CR ≥ 0.70), (3) average variance extracted (AVE ≥ 0.50), and (4) Cronbach’s alpha ( ≥ 0.70). The empirical results showed that all standardized factor loadings ranged from 0.894 to 0.976 (all significant at *p* < 0.001). Composite reliability (CR) values ranged from 0.968 to 0.974; AVE values ranged from 0.833 to 0.884; and Cronbach’s alpha coefficients for each construct were between 0.956 and 0.967. These results indicate that the measurement model demonstrates excellent internal consistency, item representativeness, and explanatory power, thereby providing strong evidence for the model’s convergent validity, as shown in Table 4.

**TABLE 4:** Convergent validity analysis of measurement model.

Construct	Item	Outer loadings	Cronbach’s alpha	Composite reliability	Average variance extracted (AVE)
Collective protective consciousness	CPC1	0.973	0.966	0.972	**0.833**
	CPC2	0.894			
	CPC3	0.902			
	CPC4	0.903			
	CPC5	0.906			
	CPC6	0.904			
	CPC7	0.905			
Intention to join emergency response team	JE1	0.972	0.956	0.968	**0.884**
	JE2	0.927			
	JE3	0.927			
	JE4	0.935			
New media engagement effect	NM1	0.970	0.960	0.969	**0.861**
	NM2	0.920			
	NM3	0.921			
	NM4	0.906			
	NM5	0.921			
Personal emergency risk perception	PE1	0.974	0.962	0.971	**0.869**
	PE2	0.915			
	PE3	0.931			
	PE4	0.915			
	PE5	0.926			
Social belongingness	SB1	0.970	0.965	0.972	**0.852**
	SB2	0.914			
	SB3	0.918			
	SB4	0.914			
	SB5	0.907			
	SB6	0.914			
Emergency response self-efficacy	SE1	0.976	0.967	0.974	**0.860**
	SE2	0.920			
	SE3	0.916			
	SE4	0.917			
	SE5	0.921			
	SE6	0.912			

CPC, collective protective consciousness; SB, social belongingness; SE, emergency response self-efficacy; PE, personal emergency risk perception; NM, new media engagement effect; JE, intention to join emergency response team.

### 4.3 Discriminant validity

This study assessed discriminant validity using the method proposed by Fornell and Larcker (1981), which evaluates the square root of the Average Variance Extracted (AVE) for each construct in comparison with its correlations with other constructs. According to this criterion, discriminant validity is established when the square root of a construct’s AVE is significantly greater than its correlations with any other construct. The data analysis results indicated that the square root of the AVE for each variable exceeded all inter-construct correlation coefficients. This confirms that each construct exhibits sufficient discriminant validity, meaning that the variables effectively capture the shared variance of their respective items while maintaining statistical distinctiveness from other constructs, as shown in Table 5.

**TABLE 5:** Discriminant validity analysis (Fornell-Larcker Criterion).

Construct	Collective protective consciousness	Emergency response self-efficacy	Intention to join emergency response team	New media engagement effect	Personal emergency risk perception	Social belongingness
Collective protective consciousness	**0.913**	**0.927**	**0.940**	**0.928**	**0.932**	**0.923**
Emergency response self-efficacy	0.639					
Intention to join emergency response team	0.589	0.629				
New media engagement effect	0.635	0.631	0.601			
Personal emergency risk perception	–0.651	–0.657	–0.644	–0.635		
Social belongingness	0.639	0.633	0.561	0.587	–0.638	

### 4.4 Model fit

The overall model fit was assessed using the Goodness of Fit (GOF) index, which is computed as a function of the geometric mean of average communality and average R^2^ value. According to Vinzi et al. (2010), a GOF value between 0.10 and 0.25 indicates weak model fit, values above 0.25 indicate moderate fit, and values > 0.36 suggest strong model fit. In this study, the GOF value was calculated as 0.670, which exceeds the threshold for strong model fit. This result demonstrates that the structural model exhibits excellent goodness of fit and strong predictive power.


$$G\,O\,F=\sqrt{\bar{A\,V\,E}}\,x\,\sqrt{\bar{R^{2}}}=\sqrt{0.860\,x\,0.522}=0.670$$

### 4.5 Path analysis

**FIGURE 2:**
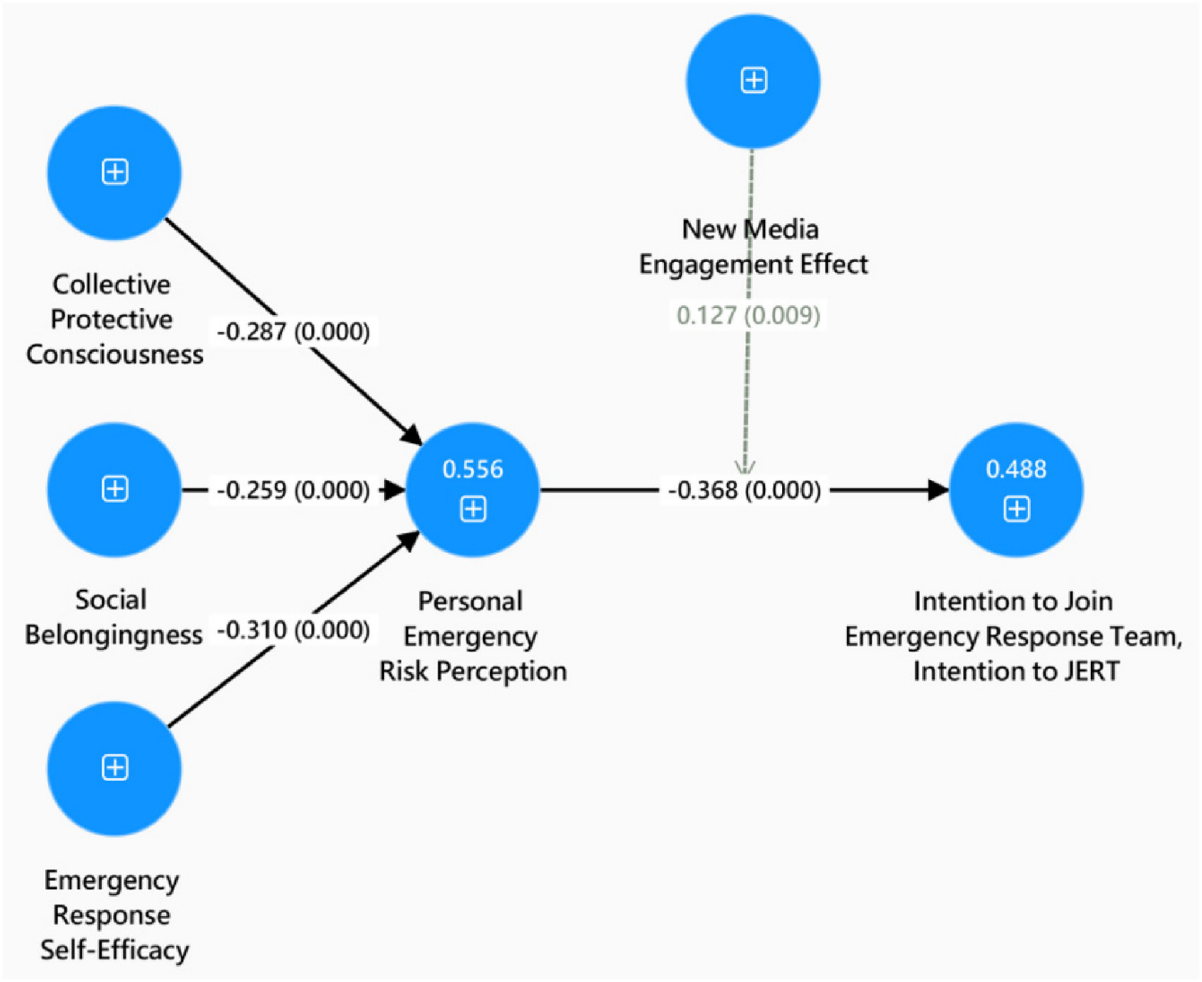
PLS-SEM structural model diagram.

This study examined the direct effects of multiple variables on Personal Emergency Risk Perception, as well as its subsequent influence on Intention to Join Emergency Response Team. The results revealed that Collective Protective Consciousness had a significant negative effect on Personal Emergency Risk Perception (path coefficient = –0.287, *t* = 5.990, *p* < 0.001). Similarly, Emergency Response Self-Efficacy was found to be significantly negatively associated with Personal Emergency Risk Perception (path coefficient = –0.310, *t* = 6.653, *p* < 0.001), indicating that individuals with greater confidence in their ability to respond to emergencies tended to perceive lower levels of subjective risk. Social Belongingness also exhibited a significant negative relationship with Personal Emergency Risk Perception (path coefficient = –0.259, *t* = 5.202, *p* < 0.001). Further analysis demonstrated that Personal Emergency Risk Perception had a significant negative effect on Intention to Join Emergency Response Team (path coefficient = –0.368, *t* = 7.164, *p* < 0.001). These results are summarized in Table 1 and Figure 2.

### 4.6 Mediation effects

This study further tested the mediating role of Personal Emergency Risk Perception in the relationships between three predictor variables and Intention to Join Emergency Response Team. The results indicated that Social Belongingness exerted a significant indirect effect on Intention to Join Emergency Response Team through Personal Emergency Risk Perception [path coefficient = 0.095, *t* = 4.194, *p* < 0.001, 95% confidence interval (0.053, 0.142)]. Similarly, Collective Protective Consciousness also demonstrated a significant mediating effect [path coefficient = 0.106, *t* = 4.541, *p* < 0.001, 95% confidence interval (0.064, 0.153)]. Among the three predictors, Emergency Response Self-Efficacy exhibited the strongest indirect effect [path coefficient = 0.114, *t* = 4.717, *p* < 0.001, 95% confidence interval (0.070, 0.164)]. These findings confirm that Personal Emergency Risk Perception serves as a key psychological mechanism in the pathway from motivation-related constructs to behavioral intention, as shown in Table 6.

**TABLE 6:** Mediation effect analysis.

Mediated path	Indirect effect (β)	SD	T statistics	*P*-values	2.50%	97.50%
Social belongingness →personal emergency risk perception →intention to join emergency response team, intention to JERT	0.095	0.023	4.194	0.000	0.053	0.142
Collective protective consciousness →personal emergency risk perception →intention to join emergency response team, intention to JERT	0.106	0.023	4.541	0.000	0.064	0.153
Emergency response self-efficacy →personal emergency risk perception →intention to join emergency response team, intention to JERT	0.114	0.024	4.717	0.000	0.070	0.164

### 4.7 Moderation effect

**FIGURE 3:**
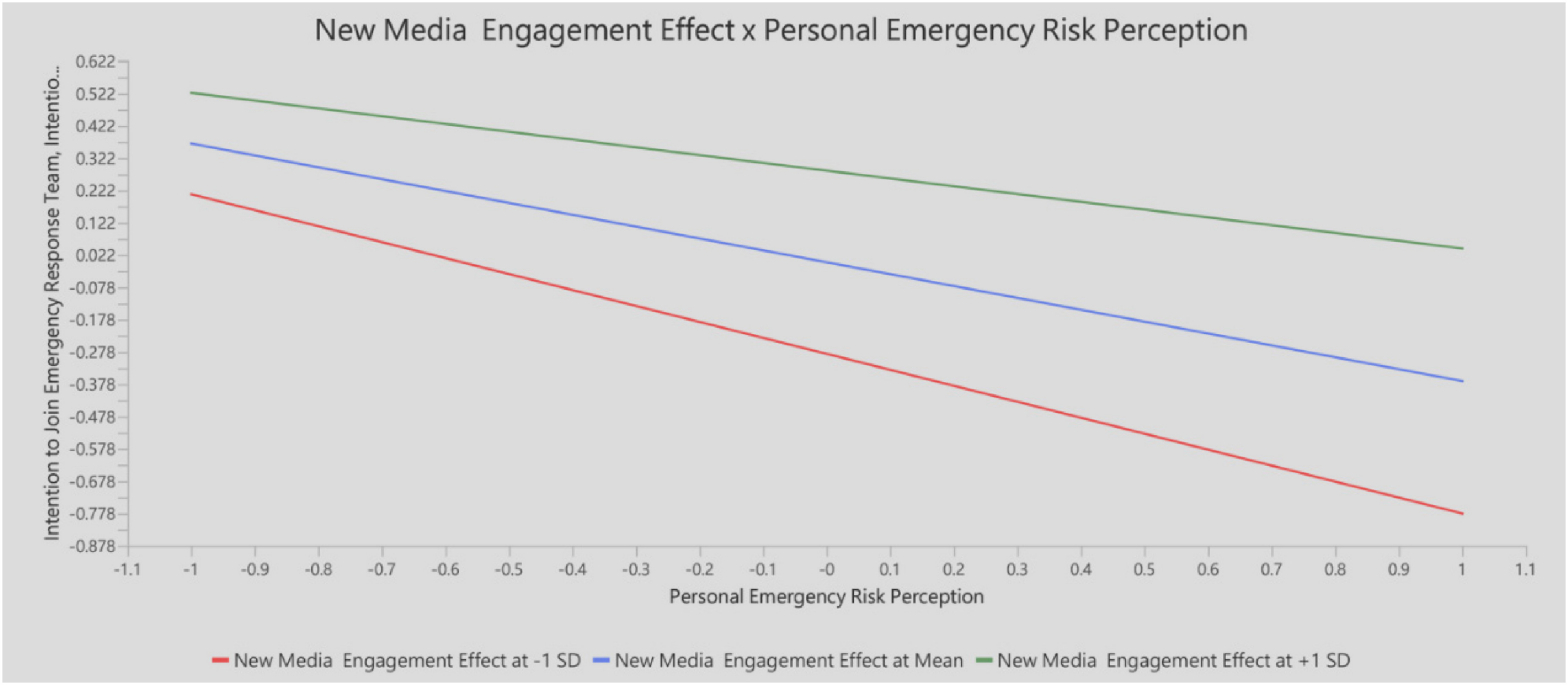
Interaction effect of new media engagement on the relationship between personal emergency risk perception and intention to join emergency response team.

This study further examined the moderating effect of the interaction between *New Media Engagement Effect* and *Personal Emergency Risk Perception* on *Intention to Join Emergency Response Team*. The analysis results indicated a significant positive interaction effect between the two variables (path coefficient = 0.127, *t* = 2.595, *p* = 0.009), as shown in Table 7 and Figure 3.

**TABLE 7:** Moderation effect analysis table.

Path relationship	Path coefficient	SD	*T*-value	*P*-value
New media engagement effect × personal emergency risk perception →intention to join emergency response team, intention to JERT	0.127	0.049	2.595	0.009

## 5 Conclusion and discussion

### 5.1 Conclusion

#### 5.1.1 The effects of collective protective consciousness, social belongingness, and emergency response self-efficacy on personal emergency risk perception

Collective Protective Consciousness had a significant negative effect on Personal Emergency Risk Perception, supporting Hypothesis H1. This result aligns with Self-Determination Theory (SDT), wherein collective protective consciousness reflects the internalization of social responsibility and corresponds to the integrated regulation level of motivation—an integration of autonomy and prosocial responsibility. When individuals possess this consciousness, it satisfies the SDT dimension of autonomy, thereby reducing their perceived risk. As a group-level construct, collective protective consciousness represents a mechanism through which individuals internalize group responsibility and collective goals, viewing risk as a shared challenge. This generates a “shared risk burden” psychological effect, which subsequently lowers perceived threat.

Social Belongingness also exhibited a significant negative influence on Personal Emergency Risk Perception, supporting Hypothesis H2. This finding indicates that when individuals experience higher levels of acceptance and connection within a group, their subjective assessment of risk in emergencies is correspondingly lower. On one hand, this outcome is consistent with the SDT concept of relatedness, which posits that strong social ties function as a psychological buffer, reducing anxiety and perceived threat in the face of uncertainty. On the other hand, a sense of belonging contributes to the development of collective resilience and a greater sense of individual security.

Emergency Response Self-Efficacy demonstrated a significant negative effect on Personal Emergency Risk Perception, confirming Hypothesis H3. This result suggests that when individuals have high confidence in their ability to respond to emergencies, their perceived threat level is notably reduced. This finding supports core propositions in Social Cognitive Theory, in which self-efficacy plays a central role in shaping behavioral judgments and risk evaluations. It is also consistent with the SDT construct of competence, which is essential for activating intrinsic motivation and regulating emotional responses. Individuals with high self-efficacy are more likely to focus on what they can control and anticipate positive outcomes, thereby reducing their perception of risk.

Synthesizing the empirical results of H1, H2, and H3, Collective Protective Consciousness, Social Belongingness, and Emergency Response Self-Efficacy all demonstrated significant negative effects on Personal Emergency Risk Perception. The strength of these effects varied slightly: Emergency Response Self-Efficacy exhibited the strongest influence, followed by Collective Protective Consciousness, and then Social Belongingness. These findings suggest that although all three factors effectively reduce perceived risk, they operate through distinct mechanisms and psychological levers.

Specifically, Emergency Response Self-Efficacy functions through an “internal control” mechanism by reinforcing individuals’ belief in their ability to manage risk. In contrast, Collective Protective Consciousness, while a group-level construct, involves a reappraisal of risk grounded in the internalization of social responsibility. Social Belongingness, on the other hand, represents affective social capital and operates more as an “emotional-buffering” mechanism that emphasizes emotional support and relational security.

From the perspective of Self-Determination Theory (SDT), the three variables correspond to the theory’s core psychological needs: Emergency Response Self-Efficacy aligns with competence, Social Belongingness corresponds to relatedness, and Collective Protective Consciousness reflects an integration of autonomy and social responsibility. Together, these constructs converge on a critical psychological mechanism: when individuals feel capable of responding, accepted by others, and aligned with collective goals in a disaster context, their perceived risk is significantly attenuated, leading to a more stable state of action readiness.

Although these psychological factors differ in hierarchical level and function, they each contribute to reducing perceived risk through distinct forms of perceived security. Their combined effect underscores the multifaceted nature of risk perception regulation in high-stress environments.

#### 5.1.2 The dual mechanism of personal emergency risk perception

The findings indicate that Personal Emergency Risk Perception exerts a significant negative effect on Intention to Join Emergency Response Team, thereby supporting Hypothesis H4. This suggests that individuals who perceive higher risks in disaster scenarios are less willing to participate in emergency response efforts. From the perspective of Self-Determination Theory (SDT), excessive risk perception may undermine individuals’ sense of autonomy and competence, leading to psychological hesitation and defensive attitudes toward participation.

Moreover, Personal Emergency Risk Perception functions as a mediating variable among the three psychological constructs and Intention to Join Emergency Response Team, confirming Hypotheses H5, H6, and H7. First, Collective Protective Consciousness indirectly influences Intention to Join Emergency Response Team through Personal Emergency Risk Perception, reflecting a psychological transformation pathway of “responsibility-driven → fear reduction → participation facilitation.” Second, risk perception serves as a conversion mechanism between emotional support and behavioral motivation. That is, when individuals feel accepted and connected within a community, they experience greater safety and perceived social support, thereby reducing fear of risk and enhancing their action intentions. Lastly, individuals with high self-efficacy are more likely to believe they can cope with risks, which lowers their subjective threat perception and increases their willingness to engage in emergency response efforts.

#### 5.1.3 The moderating role of new media engagement effect

This study confirms the moderating effect of New Media Engagement Effect on the relationship between Personal Emergency Risk Perception and Intention to Join Emergency Response Team, supporting Hypothesis H8. The findings indicate that when New Media Engagement Effect is high, the negative impact of Emergency Risk Perception on Intention to Join Emergency Response Team becomes weaker. In other words, even under high perceived risk, individuals with greater engagement in new media are more likely to participate in emergency response activities.

This suggests that New Media Engagement Effect not only serves as an information dissemination platform in emergency contexts but also plays a critical role in cognitive adjustment and emotional support. Through social interaction, emotional resonance, and experience sharing, new media enhances individuals’ perceived controllability of risk scenarios, thereby mitigating the inhibitory effects of perceived threat. Furthermore, as a medium for collective communication, New Media Engagement Effect may amplify a sense of social support and public responsibility, transforming individual tendencies toward “risk avoidance” into an “action-oriented” social response. This pathway illustrates the value of the “technology–psychology” interaction mechanism in shaping public mobilization strategies.

### 5.2 Discussion

#### 5.2.1 Theoretical contributions

Grounded in Self-Determination Theory (SDT), this study developed a three-dimensional integrated model encompassing the core pathway of “psychological motivation–risk perception–behavioral intention,” making the following key academic contributions in the field of public emergency response participation:

First, theoretical integration across domains. By introducing SDT into the context of emergency rescue behavior during public crises, this study systematically integrates the elements of intrinsic motivation, risk perception, and behavioral intention. It extends the theoretical boundary of SDT and establishes a comprehensive path model from psychological drivers to behavioral intentions, thereby addressing the theoretical gap in applying motivational psychology to collective action research. Second, innovative variable design. The study incorporates “Collective Protective Consciousness” as a group-level psychological construct, enriching the theoretical understanding of how collective awareness influences individual decision-making and behavioral intentions. This also bridges the theoretical disconnect between individual and collective mechanisms in the domain of emergency mobilization. Finally, systematic validation of the moderating mechanism of the “New Media Engagement Effect.” This study empirically explores the moderating effect of new media engagement on the relationship between personal emergency risk perception and behavioral intention. It highlights the dynamic interplay between technology, psychology, and behavioral response, thus offering theoretical support for understanding the psychological impacts of digital media in emergency response contexts.

#### 5.2.2 Managerial implications

This study explores the psychological mechanisms underlying public participation in emergency rescue based on the “psychological motivation–risk perception–behavioral intention” pathway. The findings provide the following important managerial insights for emergency management and mobilization practices:

First, stimulating intrinsic motivation is a key entry point for promoting public engagement in rescue efforts. Emergency management authorities should emphasize psychological mobilization strategies, enhance public value identification with the goal of “collective protection,” and strengthen their role perception and emotional belonging in disaster response, thereby cultivating sustainable motivation for civic participation. Second, risk communication and capacity building should be advanced in parallel. Relevant agencies must enhance community-level preparedness training and simulation drills to improve individuals’ self-efficacy, thereby transforming “risk fear” into “risk response.” Third, digital media can serve as an effective lever for social mobilization. In future emergency responses, both government and civil society organizations should leverage social media platforms to promote transparent dissemination of risk information, design emotionally resonant content and interactive mechanisms, and amplify public responsibility awareness through media influence to foster a positive public opinion environment. Lastly, it is essential to promote multi-stakeholder collaboration and establish a synergistic system integrating individuals, communities, and technology. Cross-sectoral cooperation should be encouraged, involving media platforms, community organizations, and educational institutions to achieve an organic integration of risk education, capacity empowerment, and emotional mobilization.

### 5.3 Limitations and future research directions

Although this study achieved certain theoretical and empirical contributions, it also has several limitations, which open avenues for future research: First, the sample scope and representativeness are limited. Future studies should expand the geographical coverage of the sample to enhance the generalizability and applicability of the findings. Second, the measurement of variables primarily relies on self-reported questionnaires. Future research could incorporate multi-source data, such as behavioral observation, interviews, or physiological indicators, to more accurately capture and interpret psychological constructs. Third, the study adopts a cross-sectional design, which restricts the ability to capture the dynamic evolution of causal relationships among variables. Future research may adopt longitudinal or experimental designs to trace how public psychological mechanisms shift in response to changing risk scenarios, thereby validating the stability and temporal sequencing of the proposed pathways. Lastly, the underlying mechanisms of technological factors require further refinement. Future research could apply more fine-grained models of media engagement to examine the heterogeneous effects of different forms of digital interaction on risk perception regulation.

## Data Availability

The datasets presented in this study can be found in online repositories. The names of the repository/repositories and accession number(s) can be found in the article/supplementary material.
